# On the Importance to Acknowledge Transposable Elements in Epigenomic Analyses

**DOI:** 10.3390/genes10040258

**Published:** 2019-03-31

**Authors:** Emmanuelle Lerat, Josep Casacuberta, Cristian Chaparro, Cristina Vieira

**Affiliations:** 1 CNRS, Laboratoire de Biométrie et Biologie Evolutive, Université de Lyon, Université Lyon 1, UMR 5558, F-69622 Villeurbanne, France; Cristina.Vieira@univ-lyon1.fr; 2 Center for Research in Agricultural Genomics, CRAG (CSIC-IRTA-UAB-UB), Campus UAB, Cerdanyola del Vallès, 08193 Barcelona, Spain; josep.casacuberta@cragenomica.es; 3 CNRS, IHPE UMR 5244, University of Perpignan Via Domitia, IFREMER, University Montpellier, F-66860 Perpignan, France; cristian.chaparro@univ-perp.fr

**Keywords:** transposable element, epigenomics, bioinformatic tools

## Abstract

Eukaryotic genomes comprise a large proportion of repeated sequences, an important fraction of which are transposable elements (TEs). TEs are mobile elements that have a significant impact on genome evolution and on gene functioning. Although some TE insertions could provide adaptive advantages to species, transposition is a highly mutagenic event that has to be tightly controlled to ensure its viability. Genomes have evolved sophisticated mechanisms to control TE activity, the most important being epigenetic silencing. However, the epigenetic control of TEs can also affect genes located nearby that can become epigenetically regulated. It has been proposed that the combination of TE mobilization and the induced changes in the epigenetic landscape could allow a rapid phenotypic adaptation to global environmental changes. In this review, we argue the crucial need to take into account the repeated part of genomes when studying the global impact of epigenetic modifications on an organism. We emphasize more particularly why it is important to carefully consider TEs and what bioinformatic tools can be used to do so.

## 1. Introduction

Eukaryotic genomes are much more than an array of protein-coding genes, which usually account for a small fraction of the genome space. For example, they represent only a very small fraction of the human genome (<2% of the genome), whereas repeated sequences represent more than half of it [1]. The proportion of genes is slightly larger in some organisms with a smaller genome size, such as Drosophila in which the protein-coding genes represent about 13% of the genome [2]. Historically, the non-coding part of genomes was first thought to be non-functional [3]. It is however now known to represent a mixture of repetitive DNA and non-functional sequences interspersed with non-coding RNA genes and regions that are important for transcriptional and post-transcriptional regulation [4,5]. Transposable elements (TEs), which are mobile genetic elements, account for a large part of repeated DNA and are highly diverse in structure, mechanisms of transposition, and repetitiveness in the genome. They are classified according to various features such as their intermediate of transposition (RNA or DNA) and their structural domains (enzymatic domains, regulatory sequences, non-coding repeats, etc.) or their insertion sites. On this basis, several efforts have been made to identify the main types of TEs in a comprehensive manner allowing to determine different hierarchical levels of classification going from the classes to the superfamilies (see, e.g., [6,7]). Globally, two main classes have been described: Class I comprises the retrotransposons, which contain the LTR (Long Terminal Repeat)-retrotransposons (endogenous retrovirus-like mobile elements) and the non-LTR retrotransposons grouping the LINE and SINE elements (standing for Long and Short Interspersed Nuclear Elements respectively); and Class II comprises the DNA transposons. Below the level of superfamilies, TEs are classified into families, based on sequence similarities. According to the age of a TE family and the location of its insertions, the copies may be either active and full length or degraded and transposition defective, and therefore, different copies inside a given TE family may have different degree of sequence similarity. This implies that, in some organisms, several almost identical TE copies may be present in different regions of the genome, which represents a technical barrier for the assembly of complete genomes. Moreover, the number and size of TE families are very variable according to the organisms, as well as the number of active families (i.e., families with current transposition capacity). For example, in the human genome where TEs make up 45% of the genome, which represent millions of copies [3], the most widely represented types of TEs correspond to non-LTR retrotransposons from two different subtypes (16.9% of LINEs and 10.6% of SINEs) in which only three active families are identified while the remaining ones correspond to old and more or less degraded sequences [4,8]. On the contrary, in *D. melanogaster*, TEs represent 15–20% of the genome corresponding to several thousands of copies but with a large variety of different families (~100 families especially corresponding to LTR-retrotransposons and non-LTR retrotransposons), among which the majority is still active [9,10]. TEs have a significant impact on genome evolution and on gene regulation [11,12]. Several examples have indeed shown that a large proportion of them have been recruited to provide regulatory elements allowing to generate regulatory (see for reviews [13,14,15]). TEs have also played a role in the evolution of sexual chromosomes as it has been demonstrated in different plants and animals [16,17,18,19,20,21]. However, despite the role played by TEs in the evolution of complex genomes, transposition is still a highly mutagenic event. Genomes have therefore evolved sophisticated mechanisms to control their activity, among which epigenetic modifications play an important role [22]. TEs are actually the main target of epigenetic mechanisms and they concentrate most epigenetic repressor marks in the genomes [22].

In addition to their role in the control of TEs, epigenetic modifications explain part of the variation of gene expression observed inside and between organisms. In particular, changes in gene regulation may play a role in the adaptation of organisms. For example, significant genome-wide DNA methylation differences were observed between two populations of a freshwater snail living in different habitats, suggesting that the environmentally induced epigenetic divergence was responsible for the expression of adaptive shell shape variations [23]. In human, various environmental exposures affect the epigenome and are particularly important during early fertilization and embryonic development, some of which could lead to reproductive diseases (reviewed in [24]). Thus, epigenetic modifications can be viewed as important factors to explain part of the adaptation of organisms to changing environments by providing, in particular, phenotypic plasticity.

In parallel, several cases of adaptation due to some TE insertions have been described, such as the role of LTR retrotransposon and DNA transposon insertions in insecticide resistance of *D. melanogaster* [25,26] or in adaptation to high latitudes in soybean [27]. Since TEs are the providers of significant adaptive responses of the organisms to particular environmental challenges and since both TEs and epigenetic mechanisms are linked, it has been proposed that combination of TEs and epigenetic mechanisms could allow a rapid phenotypic adaptation to global environmental changes [28,29]. The fact that some TEs are sensitive to environmental biotic and abiotic factors that can mediate their mobilization and that epigenetic modifications are also sensitive to the environment suggest that both can work together [28].

In this review, we want to underline the crucial importance of taking into account the repeated portion of genomes when studying the global impact of epigenetic modifications on an organism. However, TEs and other repeated content of genomes are often neglected when it comes to epigenomic analyses or are, at best, only partially acknowledged [30,31]. We emphasize more particularly why it is important to carefully consider TEs by providing biological examples of the importance of TEs in epigenomic studies and several examples of bioinformatic resources that can be used to do so.

## 2. Examples of the Importance of TEs in Epigenomic Analyses (à Mettre Avant)

### 2.1. In Drosophila and Other Diptera

For decades, *Drosophila melanogaster* has been a model to investigate various questions in biology and genetics. In particular, numerous works have thoroughly described its TE content, which has been estimated to represent around 15–20% of its entire genome [9,10] but only 5% when considering only the euchromatin part [32,33,34]. The availability of its genome sequence allowed showing that its TEs were mainly composed of active and newly transposed copies [9,34,35]. A first comparative approach of *D. melanogaster* with close relatives showed that, in *D. yakuba*, *D. simulans* and *D. sechellia*, TE copies are rather old and degraded [36]. Moreover, the global proportion of TEs in Drosophila genomes is highly variable. In particular, some species display a TE content largely higher than what is observed in *D. melanogaster*, for example *D. ananassae* (24.93% of repeats) and *D. suzukii* (30.80% of repeats) [32,37]. Such a variation in TE content may be the reflection of variation in epigenetic regulation at work in these related species. In other more distantly related Diptera, for which more than one hundred genomes have been sequenced to date [38], the amount of TEs may also be very variable and sometimes much larger than what is observed in *D. melanogaster*, generally associated with larger genome sizes. This is the case for example for the southern house mosquito *Culex quinquefasciatus* (genome size of 579 Mbp) [39], the mosquito *Aedes aegypti* (genome size of 1.38 Gbp) [40], and the tiger mosquito *A. albopictus* (genome size of 1.97 Gbp) [41]. These species, respectively, harbor 29%, 47% and 33.58% of TEs. However, the question of the TE activity in these species remain speculative since very few studies have tried to estimate what fraction of these TEs are actually transposing. Indeed, most studies focus on *D. melanogaster* [42,43,44,45]. Recently, some information has been provided for its sister species, *D. simulans*, showing that TE expression is variable among natural strains and that it is associated to the amount of piwi-interacting RNAs (piRNAs) [46].

Contrary to other organisms, epigenetic regulation in Diptera is mainly due to histone tail modifications and small RNAs, e.g., piRNAs, since the DNA methylation has been shown to be almost completely absent [47,48]. The chromatin conformation around genes has been shown to be influenced by the presence of TEs. In particular, euchromatic TEs can be silenced by heterochromatin formation mediated by piRNAs, such heterochromatin being able to spread to neighboring genes in Drosophila [49]. In this example, piRNA sequences directed against a DNA transposon behave as cis-acting targets for heterochromatin assembly, more specifically, the heterochromatic states of genes depend on the number and distance of nearby TEs and on the possibility for TEs to be the target of piRNAs [50]. These effects could be particularly important in the case of stress response. For example, a single insertion of a DNA transposon is associated with the up-regulation of two genes having a role in the resistance to oxidative stress [51]. This insertion is enriched in several histone modifications in stress condition when compared to the normal condition, which is consistent with the pattern of expression changes of the neighboring genes [52]. More generally, in the case of organism aging, epigenetically silenced TEs have been shown to become activated as cellular defense and surveillance mechanisms fail with age in Drosophila [53]. Indeed, transcripts of many heterochromatic genes and TEs were found to increase with age in flies, which was repressed when flies were submitted to a diet known to extend life span. Moreover, changing the expression of genes known to affect heterochromatin structure alleviated age-related expression increases of TEs. The effect of TEs in association with epigenetic modifications may also be implicated in the genome evolution at larger scale. The distribution of TEs in the genomes may be highly variable, with a huge proportion found on the Y chromosome of *D. melanogaster*, which contrasts with the few protein coding genes. However, this chromosome has been shown to be associated with natural phenotypic variations. One of the hypotheses to explain this action is the heterochromatin sink model, which suggests that variation in the heterochromatin blocks on this chromosome can be used as a sink for transcription factors or chromatin regulators, allowing for example to indirectly influence epigenetic landscapes between sexes [54,55,56,57]. TEs seem also to play a role in the formation of the Y chromosome itself and more particularly in its degeneration, as has been shown in *D. miranda* [18]. 

### 2.2. In Mammalian Genomes

In mammals, TEs represent millions of insertions but only few families are actually active and generating new insertions [8]. In normal mammalian cells, TEs are usually methylated, therefore transcriptionally silenced, which may avoid any deleterious effects. In some abnormal cells where DNA methylation is abolished, TEs can be mobilized, resulting in a potential impact on the genome stability [58,59]. In human glial tumors, a particular brain cancer, analysis of Alu element methylation level allowed determining that their level of methylation decreased when compared to a normal tissue and is more pronounced in aggressive tumors compared to nonaggressive primary tumors [60]. Furthermore, using bisulfite sequencing approach, the CpG sites with methylation loss during progression of pediatric intracranial ependymomas allowed generating methylation profiles for thousands of Alu elements and their flanking sequences. When compared, the methylation profiles between normal and tumor tissues showed an unchanged methylation status of a majority of CpG sites adjacent to or within Alu elements. Thus, by their study, the authors proposed that the methylation status of specific Alu elements could serve as prognostic factors for some brain cancers. Another study analyzed Alu element methylation profiles in colon cancer and normal tissue [61]. It allowed showing that normal cells display a low proportion of unmethylated Alu elements that increases up to 10-fold in cancer cells. Interestingly, in normal tissues, hypomethylated Alu elements are located near functionally GC rich regions and display other epigenetic features consistent with an impact on genome regulation. In cancer cells, when considering different regions of the genome according to the high or low rates of Alu element hypomethylation, it was possible to identify genomic compartments with differential genetic, epigenetic, and transcriptomic features, associated with higher chromosomal instability.

The DNA methylation is also known to be particularly important in embryonic development of mammals. The analysis of human preimplantation embryos allowed showing that the genome is globally demethylated. However, variations exist concerning TEs. Young LINEs and SINEs present a lower demethylation level than older ones, indicating that early embryos retain methylation for younger and thus more active TEs, probably to prevent too many deleterious effects due to their potential mobilization [62]. Recently however, it has been shown that the gradual loss of DNA methylation that occurs in embryonic stem cells of mouse is associated with a global reactivation of TEs but that another mechanism then compensates DNA demethylation to prevent TE mobilization through a redistribution of repressive histone modifications [63]. In another work, loss of DNA methylation in embryonic stem cells allowed the identification of an endosiRNA-based mechanism involved in the suppression of TE transcripts, suggesting that this mechanism could be an “immediate” response to global methylation erasure, which would then be followed by a “long-term” repressive chromatin response [64].

These different examples show that the study of epigenetic regulation of TEs is important to better understand what is happening during the evolution of some diseases but also during the development.

### 2.3. In Plants

Plants are sessile organisms and this makes the need to adapt to a changing environment particularly striking for them. The ability to respond to environmental stimuli depends on the capacity to regulate transcription in a precise way. Transcriptional regulation is the result of the concerted action of transcription factors but also of epigenetic changes of DNA and chromatin.

The importance of epigenetic regulation in plants was recognized even before the molecular basis of epigenetics was established. Indeed, several epigenetically-related phenomena, such as imprinting and paramutation were described in plants more than 50 years ago [65,66]. At the same time, and also working on plants, Barbara McClintock proposed the existence of mobile elements and described them as controller elements able to rearrange and regulate the genome [67,68,69]. Since then, the study on plants has contributed important concepts to the epigenetics field and has shown that epigenetic regulation of plants shares many principles with that of animals, but also presents some particularities [70]. In plants, DNA methylation not only occurs in cytosines located in a symmetric context (CG or CHG), but also happens in asymmetric contexts (CHH), which requires de novo methylation after each DNA replication round [71]. De novo methylation in plants requires the RNA dependent DNA methylation (RdDM) pathway where siRNAs of 24 nt guide the methylation of specific targets [72]. These 24 nt siRNAs are the most abundant type of small RNAs in plants. In addition to DNA polymerases POL 1–3, plants have two additional DNA-dependent RNA polymerases, POL IV and POL V, which are essential for RdDM. Pol IV generates RNAs corresponding to TEs and repetitive regions that are processed into 24 nt siRNAs and target complementary chromatin-bound RNAs produced by Pol V, which directs de novo methylation of these regions [72]. Most 24 nt siRNAs and POL V recognized regions correspond to TEs.

Plants are the main source of food for humans, and consequently humans have strongly modified plants through breeding since the Neolithic times to obtain new plant varieties better suited for human needs. Both genetic and epigenetic variability underlay the phenotypic variability that is the basis for crop breeding [73]. Several epialleles conferring new traits important for crop breeding have been described, including epialleles conferring non-ripening tomatoes, producing rice varieties resistant to fungal pathogens, or controlling sex in melon [73].

TEs are the major target of epigenetic silencing, and their distribution in the genome closely parallels that of DNA methylation and repressive chromatin modifications. Silencing of TEs is highly efficient in plants and most TEs are only active in situations in which silencing is blocked (e.g., in mutant backgrounds) or alleviated (e.g., under stress) [74,75,76,77,78]. The relationship of stress and TE activation in plants was already proposed by McClintock [67,68,69], and over the years many examples have accumulated of plant TEs activated by stress [79]. In some cases, an analysis of the TE’s promoter has allowed to identify stress-related *cis*-regulatory motifs [80,81]. The combination of the presence of stress-responsive elements in the promoters of plant TEs and the alleviation of their silencing in stress situations probably explains why stress seems to be the major activator of plant TEs.

The activation or the silencing of a TE can influence the expression of the genes located nearby. In the case of LTR retrotransposons, which contain promoter sequences within the two LTRs, an insertion close to a gene can place it under the control of the element’s 3′ LTR. This is, for example, what happens in blood oranges, where the 3′ LTR of a retrotransposon inserted upstream of a MYB transcription factor of the anthocyanin pathway confers cold-stress associated activation to this gene [82]. Other types of TEs, such as Miniature Inverted-repeat Transposable Elements (MITEs), also contain transcription factor binding sites, and it has been proposed that their movement could allow rewiring new genes into transcriptional networks during evolution [83]. However, in addition to the potential effect of TE promoters on genes located nearby, TEs can influence the expression of neighboring genes through epigenetic mechanisms. Indeed, as already said, TEs are the main target of epigenetic silencing, and the deposition of repressing epigenetic marks on TEs can affect the expression of genes located nearby. Actually, most plant epialleles described so far are linked to the polymorphic presence of TEs [22,84]. A classic example is the methylation of a SINE element insertion close to the *FLOWERING WAGENINGEN* (*FWA*)-locus in Arabidopsis that results in the imprinting of this locus [85].

In the last few years, many examples of TEs influencing the expression of genes located nearby in plants have been reported and it has become clear that the epigenetic regulation of TEs greatly contributes to plant phenotypic diversity and adaptation [86]. Interestingly, although most TEs do not show a strict target specificity for integration, some families seem frequently present close to genes, and, in particular, to genes controlling environmental responses [87], and it has very recently been shown that *Copia* retrotransposons seem to target those genes for integration guided by the presence in their promoters of the histone variant H2A.Z [88].

As a summary, TEs are strongly repressed by epigenetic silencing in plants, but this repression is alleviated in some stress circumstances allowing them to move, which makes them stress sensors. As these elements seem to frequently insert close to genes, influencing their expression, they can rewire new genes to the stress-related transcription networks. This potential to alter plant gene responses to stress, and therefore their capacity to adapt to new environments, makes TEs a promising tool for generating phenotypic diversity, and strategies to transiently inhibit TE control are now been proposed as crop improvement strategies [89].

### 2.4. The Case of Schistosoma, a Non-Model Organism

Schistosoma is a parasitic flatworm and causative agent of schistosomiasis, a disease that affects over 200 million people worldwide. The parasite has a complex life cycle involving two consecutive obligate hosts (a poikilotherm snail and a homeotherm mammal) and two transitions between these hosts as free-swimming larvae (miracidia and cercariae). The parasite’s sex is determined by the presence of sex chromosomes, ZZ for males and ZW for females. With the availability of the assembled draft genome [90] and the production of a massive parallel sequencing, the “repeatome” of *Schistosoma mansoni* was assembled, consisting of 5420 sequences and estimated to represent 47% of the genome, of which 20% corresponds to TEs while the rest encompasses short repeats and microsatelites [91]. The most abundant TE classes were Class I (~66% corresponding to LTR and non-LTR retrotransposons) and Class II (24% DNA transposons).

The impact of TE presence was further analyzed. In particular, the introduction of the parasite into the American continent during the colonization allowed Wijayawardena et al. [92] to compare the Old World and New World genomes to test for the breakdown of TE repression systems after a physiological stress. They tested the abundance of TEs in New World and Old World Schistosoma by evaluating the copy number of six TEs and found that, as expected, the New World Schistosoma presented more copies of Class I and Class II elements. Interestingly, this difference was not reflected in the transcriptome suggesting that either the repression system was reactivated or the copies were all truncated. These results show that TEs are subjected to control mechanisms very similarly to other model organisms. To understand the impact of TEs on gene regulation, Philippsen and DeMarco [93] studied the distribution of TEs throughout the gene structure and observed that it was not uniform, with introns on the 3′ side of the gene showing more TE insertions when compared to the 5′ introns. Through the analysis of transcriptomic data, they found that some genes showed transcriptional variants due to the influence of inserted TEs. These elements might play a role in changing the splicing sites of genes. They also showed that six genes presented TE sequences in their coding regions, which indicates a possible domestication of the included sequence. These results highlight the importance of TEs in shaping the evolution of the Schistosoma genome and to what extent TEs have a direct impact on the gene landscape.

Once the existence of TEs was established, the next question was to elucidate in what measure these elements impacted the chromatin or epigenomic landscape of the genome. Previously, it was considered that *S. mansoni* lacked detectable DNA methylation patterns [94]. It was not until 2013 that cytosin methylation, in the CpG context, was described and found to be a conserved epigenetic feature in Platyhelminthes including the three most studied schistosomes: *S. haematobium*, *S. japonicum* and *S. mansoni* [95]. Since there are several indications that repeat elements play an important role in the chromosome structure and therefore in the chromatin landscape of the parasite, the question of the association of TEs and epigenetic modification in Schistosoma needed to be investigated. It was observed that histone methylation of TE rich regions changed during the transition from cercaria, the free-swimming larvae that infects the mammalian host to adult, which occurs inside the host [96]. These changes included the decrease of H3K9me3 and H3K27me3 enrichment as well as the increase in enrichment of H3K9ac and H3K4me3 modifications. The authors proposed that this could be an indication of large-scale chromatin rearrangements or chromosome displacements in the nucleus during transformation into adults. Moreover, they also suggested that the repeat containing large pericentromeric regions become heterochromatic when cercariae develop inside the snail. Another peculiar observation during transformation from cercariae to adults was the nearly complete loss of H3 modifications in 22 of the 36 W chromosome-specific (sex specific) repeats. It was previously shown that transcription of these repeats can only be observed during larval stages and was undetectable in adults [91]. When comparing cercariae to the adult stage in the vertebrate host, H3K27me3 was found to be differentially enriched and a high sex-biased expression of TEs, mainly those contained in introns, was observed. This work suggests that TEs could be involved in sex chromosome inactivation as well as in the dosage compensation mechanism [97]. At the developmental level, it has been shown that the different stages of the parasite can be discriminated by looking at five chromatin modifications (i.e., the chromatin landscape) throughout the complete life-cycle of the parasite and specifically through the analysis of histone methylation levels of repetitive elements. The only stages that could not be readily discriminated were the miracidia and sporocyst I stages, which are very similar [96].

As more knowledge accumulates on the importance of repeat elements, a detailed analysis on the different classes of TEs would shed a clear light of the full importance of these elements in the chromatin conformation and their potential implication in the regulation and shaping of the Schistosoma genome.

## 3. Bioinformatic Analysis of TE Sequences in Genomes

### 3.1. TE Insertion Identification and Polymorphism

The great diversity in structure, mechanism of transposition and repetitiveness of TEs makes their accurate annotation in complete genomes challenging, which is further complicated by progressive loss of sequence identity in decaying elements. The degree of complexity of this task will depend on each genome [98], as the TE population dynamics and evolutionary forces leading to different rates of TE family birth and extinction will vary from genome to genome [99]. There are two main approaches to identify TEs in genome sequences: homology-based approaches, which use sequence similarity to already characterized TEs, and de novo approaches. The sequence diversity of TEs limits the former, although similarity among coding conserved domains of enzymatic activities commonly encoded by TEs can be used to detect these regions. The de novo approaches are usually based on the repetitiveness of TE sequences or their structural motifs (e.g., the presence of LTRs for LTR-retrotransposons). Several bioinformatics tools are available based on these different approaches [7,98], and there are also pipelines that combine the different tools to be able to make a comprehensive annotation of TEs, such as the REPET package [100] (Table 1). Another tool called PiRATE has been developed recently, which includes several available packages for TE identification and annotation like similarity based and de novo structural based tools, repeat based and TE reconstruction from short reads [101]. Although these tools are in general very efficient, it is still challenging to compare the TE landscapes of different genomes only based on their TE annotations, even when they have been produced using the same tools and parameters.

The annotation of TEs in reference genomes is only a snapshot at a given time of a particular individual which may not be representative of all individuals of the species. Although a majority of TEs are no longer able to transpose, and most insertions are likely to be fixed, mobile TEs are at the origin of an important part of the genetic variability within populations. For example, in human, several thousand polymorphic TE insertions were identified when comparing 185 samples in three major populations [102]. It is therefore essential to be able to identify non-reference TE insertions. There are different bioinformatic methods available to use NGS data on individuals or varieties to identify TE insertion variability. Most of these methods use a reference genome of the species to map de NGS paired-end short reads and identify those that map discordantly, selecting those for which one of the reads maps to a previously identified TE. Examples of these include software packages such as Jitterbug [103], TEMP [104], MELT [105], PopoolationTE2 [106] or TEFLoN [107]. Others on the contrary map first to the consensus sequence of previously identified TEs and map afterwards the paired read onto the genome, such as TRACKPOSON [108]. A more recent approach using long Pacbio reads has also been reported, but it still relies on the good annotation of reference TE sequences [109]. Another approach infers insertion events using the misalignment and split alignment of short reads without relying on a database of previously identified TEs. Several of these tools have been reviewed in [110].

For non-model species, due to the general lack of a good quality genome assembly or in the case that no reference genome exists at all, the search for TEs is especially challenging. Similarity or motif-based searches will identify older elements in the assembled draft genome or transcriptome, but new elements with high sequence conservation will not be assembled and the data will be hidden in the unassembled reads. In this case, TEs have to be reconstructed from the non-assembled short reads by using software packages such as RepARK [111], Tedna [112], RepeatExplorer [113], or dnaPipeTE [41].

### 3.2. Methods to Analyze Epigenetic Modifications Associated to TEs

#### 3.2.1. On the Difficulty to Consider TE Sequences

Currently, very few tools are dedicated to specifically study epigenetic modifications associated to TEs (Table 1). Usually, these sequences are rather ignored, their repetitive nature making it difficult in some cases to assign reads produced by NGS technique to individual TE copies, which results in multi-mapping problems. As individual TE copies usually contain specific SNPs, a fraction of the reads will map to individual TE sequences unambiguously, which may be enough in some cases to draw conclusions. However, this will depend on the repetitiveness and the sequence variability (and therefore the age) of each particular TE family. Currently, the different read mappers have various ways to handle multimapping reads. For example, the default parameters of Bowtie 2 allow searching for multiple alignments and to report the best one [114]. Additional parameters may be implemented to keep uniquely mapped reads, which in this case will remove almost all information concerning TEs. Alternatively, it is possible to get all possible positions of reads or to return up to a given number of alignments. The mapping may be performed on consensus TE sequences, which represent a given TE family. However, the variation of quality among copies of a given family may have a direct impact in the process of building the sequence reference (consensus) of the considered family. These consensus sequences are usually supposed to correspond to full-length and active sequences or to represent the ancestral element at the origin of all copies currently present in a genome. Such type of sequences is available in the TE database Repbase [115], a resource that is widely used for TE annotation. Normally, only nearly full-length and thus potentially active copies are considered in the process of consensus construction. However, a consensus sequence may be quite divergent from older copies. Moreover, not all species are represented in this database, which may lead to consider consensus TE sequences from related species instead of real TE representative of the analyzed species. This may lead to a wrong assessment in TE recognition by mapping approaches but also to a loss of information from the unmapped reads. Thus, the crucial step of mapping reads on TE sequences remains a challenge that will become easier as the sequenced reads become longer.

#### 3.2.2. Histone Modifications

In the original study describing the ChIP-Seq method, to determine the chromatin landscape associated to TEs in pluripotent and lineage-committed cells from mouse [116], reads were directly aligned to a library of repetitive element consensus sequences obtained from Repbase. Since this pioneer work, other studies have used a similar approach to determine histone modifications associated to repeats. For example, to determine the chromatin landscape at centromeric and telomeric positions in the human genome, researchers mapped reads from ChIP-seq data to consensus sequences of TEs known to be specifically found in these regions [117]. Other works have tried to circumvent the problem of mapping on consensus sequences. Among them, one proposes a tool, Repeat Enrichment Estimator available as a web interface, specifically to study histone modification enrichment of repeats [118]. This tool aims at performing enrichment analyses of repeats taking advantage of their increased sequence coverage. An advantage of this method is that it incorporates both ambiguously and uniquely mapped reads to avoid bias due to reads mapping on consensus sequences. This obviously allows obtaining more informative reads associated to a given family. However, the final results are not copy specific. This method only allows obtaining histone enrichment at the family level. Another limitation is that the web interface only allows the use of a very limited number of model species (human, mouse and Drosophila). A similar approach not implemented in a comprehensive tool has been proposed in a study of histone modifications of human TEs [119]. In this work, TE insertions from the genome are used rather than consensus sequences. In this procedure, the reads are aligned on the genome and randomly assigned to a genomic location when a given read presents more than one alignment with best scores. By crossing all the locations with read assignments to coordinates of the TE annotations obtained via RepeatMasker [120] (http://www.repeatmasker.org), the authors determined the enrichment of specific TE types. Similar to the Repeat Enrichment Estimator tool, the enrichment obtained is not given at copy level and thus the information concerning the genomic location is lost. Among the set of pipelines proposed in the method piPipes, which is dedicated to piRNA and TE analysis [121], one is dedicated to the analysis of ChIP-Seq data and proposes two methods to consider multi-mapping reads. Either the best alignments of multi-mapping reads are randomly assigned to a location or the CSEM algorithm is used to assign unambiguous reads. By these approaches, it is thus possible to consider repeat regions in the global performed analyses.

#### 3.2.3. DNA Methylation

Several approaches for genome-wide methylation analysis have been developed [122]. Among them, two well-known methods, bisulfite sequencing (BS-Seq) and methyl-DNA immunoprecipitation sequencing (MeDIP-Seq), allow genome-wide analyses of DNA methylation by high throughput sequencing [123]. BS-Seq is often considered as a gold standard for high-resolution DNA methylation profiling. It is however sometimes difficult to correctly map short reads obtained from bisulfite converted genomic DNA when they derive from repeats. Other strategies have been developed to more specifically analyze methylation level of repeats. Xie et al. [124] proposed a method to amplify a large set of repeats along with their flanking sequences, from bisulfite converted genomic DNA. They were able to apply this method on Alu elements in normal human tissue. In this approach, Alu element insertions from the human genome sequence as well as consensus sequences of Alu elements from Repbase are submitted to an in silico bisulfite treatment. Specific primers from a particular subset of Alu elements are designed among these sequences to produce a specific library that will be sequenced to obtain position specific sequences. The advantage of such an approach is that it allows access to the position of methylated TEs. However, it suffers some pitfalls, e.g., it is biased toward young Alu elements that are rich in CpG; it does not take into account a potential insertion polymorphism that may occur between the reference genome and the analyzed tissue; and it only focuses on one type of TEs. Another approach has been proposed to compute methylation level on repeats using single-base DNA methylation profiles data [125]. Levels of methylation are computed on the whole genome sequence. Then, the position of TE insertions with at least one CpG are recorded from TE annotations from the human genome reference. In their work, the authors group the different TEs in large classes (SINE, LINE, etc.) to obtain global methylation levels for each category. As the individual TE copies usually contain specific SNPs, some reads may map to TEs unambiguously. Actually, many analyses of TE methylation at the genome level are done with uniquely mapped reads only. Even if it is still possible using such an approach to get information of the methylation level for an individual insertion, the insertion polymorphism between the sample and the reference genome is not taken into account, which would prevent confidently observing individual insertion methylation level. More recently, a method called High-Throughput Targeted Repeat Element Bisulfite Sequencing (HT-TREBS) has been developed with the aim to determine which individual repeat elements escape methylation repression, since they may be the ones with a direct impact on the genome [126]. In this method, specific primers are designed to study particular TEs, and regions flanking these insertions are also sequenced, allowing to compare variation in the number of repeats that are or not methylated across several samples. Even if this method remains at a general level, it allows intra- and inter-individual comparisons. However, the element specific primer approach implies that not all TEs can be considered in the same analysis. To try to circumvent the drawbacks of these methods and in the perspective to detect only unmethylated Alu elements in human cancer conditions, Jorda et al. [61] developed the Next-generation Sequencing of UnMethylated Alu (NSUMA) method to selectively enrich for DNA fragments composed of an unmethylated Alu repeat and its flanking regions, allowing an exact mapping in the genome. A ratio of differential DNA methylation of Alu elements can then be calculated among different samples. Having the sequence of the flanking region also allows to determine the position in the genome even in the case of polymorphic insertions. This method allows a reduction in the sample complexity and the unique mapping of the considered repeat. However, this method is focused on a very particular type of human TEs but it probably may be possible to consider its adaptation to other types of TEs. Recently, a new bioinformatic method, EpiTEome, has been proposed with the simultaneous goal to detect both TE insertion sites and their DNA methylation level [127]. This approach allows detecting, in addition to known TE insertions, any new insertions. Using BS-seq data, this program first retains discordant reads with one mapping an annotated TE. Then, by a split-read approach, the breakpoint location of the TE insertion is identified. Simultaneously, the methylation status of the insertion and the flanking region is determined. Although this tool represents a great advance, the authors pointed out some limitations on which further developments need to be done, e.g., the necessity of a high sequencing coverage and having a closely related reference genome, and the fact that nested TE insertions will not be found.

#### 3.2.4. Small RNA Regulation

The third epigenetic mechanism having an impact on TE activity is represented by the action of small RNAs (sRNAs). This regulation system is particularly important to control TEs in Drosophila. To study this mechanism, it is also essential to take into account at the same time the expression level of TEs. A first approach was proposed to analyze specific sRNAs of Drosophila from RNA-seq data [128]. However, this method leads to a loss of information since only reads mapping at unique positions with no mismatch on a reference genome are considered as well as using consensus sequences of TEs to associate sRNAs to a TE family. Such a method is currently implemented in the piPipes pipeline [121], which is suited only to analyze well annotated reference genomes. To take into account these drawbacks, a new pipeline, TEtools, has recently been proposed [46]. This tool has been developed for the analysis of TE expression for both mRNAs and sRNAs. It takes into account the TE sequence diversity of a genome and can thus be applied even on unannotated or unassembled genomes as long as TE sequences have been identified. It thus performs the mapping of RNA-seq data directly on TE sequences and not on the complete genome, and performs differential expression analyses when several samples are given. More recently, a new tool, SQuIRE has been proposed to analyze the TE expression level in a locus specific manner allowing to assess variation of expression between the different copies of a given family [129].

#### 3.2.5. Prospective Issues

Methodological efforts are still needed to study epigenetic modifications directly associated to TE sequences, even if some tools and methods already exist. It is clear that progress needs to be made, especially in the association of chromatin modifications and individual TE insertions. The majority of the developed tools are also largely biased toward specific species, especially mammals. It is thus necessary to develop more polyvalent tools that could also work for non-model species. A prerequisite is to try as much as possible to rely on individual TE insertion sequences rather than on TE consensus. It is particularly important to consider the global sequence diversity of a TE family that may be very variable but also the variable genomic environment of a given insertion that may have consequences on the associated epigenetic modifications. The main difficulty in handling repeats in such analyses clearly comes from their repetitive nature that makes the unambiguous mapping of the short NGS reads challenging. Currently, the size of the reads is smaller than a complete TEs, especially in the case of ChIP-Seq data. This size is important to allow discrimination between similar sequences as well as to have access to their localization (via overlapping reads between flanking regions and TE sequences). It could be considered to directly assemble reads to artificially obtain longer reads as a first step to bypass small read size. However, as long read technologies will continue to be developed, such a problem should be easier to handle in a close future. To take into account the population genomic dimension of some analysis, a possibility to be considered could be to put an emphasis on the identification of TE flanking regions, as it is performed by insertion polymorphism identification tools.

## 4. Conclusions

TEs have an important role in genome functioning and evolution mainly because of the large variety of mutations they can generate, which can have an impact on the response of the organism to environmental changes. TEs are also the main target of various epigenetic mechanisms, mainly to silence them and control their activity. These epigenetic mechanisms are also considered to adjust or generate new phenotypes, associating the living environment of organisms with their genomes and phenotypes. It is thus clear that both TEs and epigenetics mechanisms play an important role in organism adaptation, independently but also jointly, making it crucial to consider TEs, along with other repeats, when analyzing globally any epigenome.

## Figures and Tables

**Table 1:** Bioinformatic tools allowing the identification and analysis of TEs.

Usage	Tool	Question	References
TE annotation in assembled genomes	REPET	Pipeline of tools based on “all-by-all” blast search to detect and annotate repeats in genomic sequences	[100]
	PiRATE	Pipeline combining multiple analysis tools representing all the major approaches for TE detection	[101]
TE polymorphism detection	Jitterbug	Identifies new TE insertions in a sample with respect to a reference genome and predicts the allelic frequency of the insertions	[103]
	TEMP	Identifies both the presence and absence of TE insertions in genomic DNA sequences	[104]
	MELT	Identifies TE insertions on a population scale; Developed on the 1000 Human genomes project data	[105]
	PopoolationTE2	Identifies both new and annotated TE insertions allowing the comparison of TE abundance among pooled population samples or different tissues	[106]
	TEFLoN	Identifies the breakpoints and superfamily identity of both new and known TEs	[107]
	TRACKPOSON	Identifies TE insertions in large dataset based on known TE consensus	[108]
	LoRTE	Uses PacBio long read sequences to identify TE deletions and insertions between a reference genome and other sampled genomes	[109]
TE annotation in unassembled genomes	RepARK	Produces the assembly of de novo repeat sequences from detected repeat k-mer directly from raw reads.	[111]
	Tedna	Assembles TEs directly from the reads	[112]
	RepeatExplorer	Pipeline to identify and characterize repetitive DNA elements from NGS data using a graph-based clustering approaches to identify repeats, and additional programs to annotate and quantify them.	[113]
	dnaPipeTE	Pipeline to de novo assemble, annotate and quantify repeats from NGS low coverage genomic datasets.	[41]
Epigenetic analysis of TEs	Repeat Enrichment Estimator	Histone modifications associated to TE families; Web interface developed to analyze human, mouse and Drosophila data.	[118]
	piPipes	Set of pipelines to analyze histone modification, piRNAs and expression level associated to TEs	[121]
	EpiTEome	Simultaneous goal to detect both TE insertion sites and their DNA methylation level.	[127]
	TEtools	Analysis of TE expression for both mRNAs and sRNAs, taking into account the TE sequence diversity.	[46]
	SQuIRE	Analysis of the TE expression level in a locus specific manner to assess variability between the expression of the different copies of a given TE family.	[129]
